# MicroRNA-mediated changes contributing to benzo[a]pyrene toxicity in a 3D respiratory model for asthma

**DOI:** 10.1016/j.crtox.2026.100283

**Published:** 2026-01-22

**Authors:** Reese M. Valdez, Yvonne Chang, Jamie M. Pennington, Susan C. Tilton

**Affiliations:** a Environmental and Molecular Toxicology Department, Oregon State University, Corvallis, OR, USA; b Superfund Research Center, Oregon State University, Corvallis, OR, USA

**Keywords:** Benzo[a]pyrene, Human bronchial epithelial cells, microRNA, Asthma, Interleukin-13

## Abstract

There is increased emphasis on understanding how non-chemical stressors that contribute to inflammation in the lung may influence adverse health outcomes after chemical exposures. Prior studies in an *in vitro* respiratory model of type 2 asthmatic inflammation found cells from the asthmatic phenotype respond uniquely to benzo[a]pyrene (BAP) treatment compared to normal cells across multiple endpoints related to mucus production, goblet cell hyperplasia, mucociliary dysfunction and airway remodeling. To further understand how cellular response to BAP is regulated in a model of inflammation-based disease, this study examines changes in miRNA and mRNA regulation following BAP exposure in primary human bronchial epithelial cells (HBECs) cultured at the air–liquid interface with normal and interlukin-13 (IL-13) induced asthmatic phenotypes. Primary 3D HBECs differentiated in the presence and absence of 10 ng/mL IL-13 were treated on day 25 with 158 µM BAP for 48 h. Differentially expressed (q < 0.01) miRNA and mRNA were analyzed to predict miRNA target interactions and assess the functional consequences of miRNAs in each phenotype. While BAP-treated HBEC with the IL-13 asthmatic phenotype had a similar number of differentially expressed miRNA (93 up- and 100 down-regulated) compared to BAP-treated normal HBEC (93 up- and 94 down-regulated), IL-13 HBEC treated with BAP were shown to have unique enrichment of miRNA targets involved in up-regulation of cell cycle processes and down-regulation of processes related to NOTCH, WNT, and Hedgehog signaling. These data are the first to provide insight into the role of miRNAs as regulators of chemical toxicity in a respiratory model of inflammation-based disease.

## Highlights

• The IL-13 induced asthma model exhibited unique miRNA-mediated response to BAP compared to the normal model.
• Notch, Wnt, and Hedgehog signaling processes were uniquely targeted by miRNA in the BAP treated IL-13 induced asthma model.
• miRNA-mediated dysregulation of targets engaged in cell cycle processes resulted in unique risk in the BAP treated IL-13 induced asthma model.

## Graphical abstract

**Figure:**
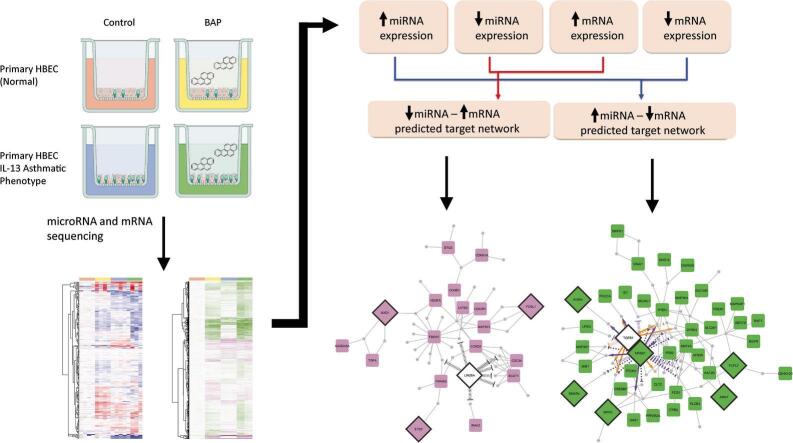

## 1 Introduction

Risk to vulnerable and susceptible populations in response to chemical stressors is not fully understood due to the existing knowledge gaps for how chemical toxicity contributes to adverse health outcomes in these individuals (Rider et al., 2014, Sexton, 2012). Prior work from our lab and the published literature show that pre-existing pulmonary inflammation alters how cells respond to polycyclic aromatic hydrocarbon (PAH) exposures potentially increasing susceptibility for toxicity and disease (Valdez et al., 2024, Arlt et al., 2015). While it has previously been demonstrated that exposure to PAHs and other environmental contaminants are associated with development and exacerbation of chronic airway diseases, like asthma and chronic obstructive pulmonary disorder (COPD), little is known about how the presence of these diseases alters toxic response to chemical insult and the mechanisms involved (Montano et al., 2025, Nwaozuzu et al., 2021, Låg et al., 2020).

PAHs are a large class of chemicals made of up of fused benzene rings that are ubiquitous environmental contaminants typically formed through incomplete combustion from natural or anthropogenic sources such as forest fires and cigarette smoke, respectively (Abdel-Shafy and Mansour, 2016). Several PAHs have been identified as chemicals of concern by the US EPA due to their prevalence and risk of adverse health effects, including benzo[a]pyrene (BAP), which has also been classified as a Group 1 carcinogen by IARC (Jameson et al., 2019). BAP exposure is associated with various cancers, including lung cancer, particularly after the chemical has been metabolized (Abdel-Shafy and Mansour, 2016). Outside of cancer endpoints of toxicity, BAP and other PAHs have been found to contribute to non-malignant respiratory outcomes (Låg et al., 2020). A wide variety of non-cancerous health outcomes are associated with BAP and other PAHs, such as oxidative stress and disruption of the lung epithelial barrier, suggesting that respiratory effects of PAHs are not limited to carcinogenesis alone (Låg et al., 2020).

Asthma is a major non-communicable chronic disease that affects children and adults (World Health Organization, 2024). Globally, asthma is estimated to affect over 260 million people with prevalence rates varying from country to country. In the United States, nearly 8% of the population has asthma (Centers for Disease Control and Prevention, Most Recent National Asthma Data | CDC, 2024). The disease is characterized by airflow obstruction caused by inflammation and presents with other symptoms like mucus hypersecretion and airway hyperresponsiveness; however, it is also a complex disease with various endotypes and phenotypes (Dharmage et al., 2019, Gans and Gavrilova, 2020). The disease is broadly separated into two major endotypes: T2 high and non-T2. T2 high asthma, which is characterized by high levels of type 2 inflammation in the airways, encompasses several asthmatic subtypes accounting for the highest proportion of asthma cases (Ricciardolo et al., 2021, Kuruvilla et al., 2019). Type 2 inflammation in T2 high asthma is primarily driven by several interleukins (IL), predominately IL-13 (Maspero et al., 2022). IL-13 has been shown to result in an increase in mucus-producing cells and a decrease in ciliated cells resulting in airway damage and obstruction (Laoukili et al., 2001). This response contributes to mucociliary dysfunction as well as other hallmark characteristics of type 2 inflammation (Maspero et al., 2022).

Previous studies have shown that IL-13 can be used in *in vitro* airway epithelial cell models cultured at the air–liquid interface (ALI) to generate asthma-like phenotypes (Valdez et al., 2024, Laoukili et al., 2001, Mertens et al., 2017, Jackson et al., 2020). When cultured in the presence of IL-13, airway epithelial models exhibit signs of goblet cell hyperplasia, increases in mucus production markers such as *MUC5AC*, and decreased barrier integrity. These characteristics are consistent with type 2 inflammation associated with T2 high asthma. In recent years, interest in using organotypic cultures and differentiated cell culture systems as alternatives to animal models has grown due to their ability to recapitulate the structure and function of the airway *in vivo* (Rayner et al., 2019, Bedford et al., 2022). Furthermore, primary cell cultured at the ALI have been found to better mimic *in vivo* airway responses compared to immortalized cell lines cultured at the ALI (Pezzulo et al., 2011).

MicroRNA (miRNA) play a significant role in regulating disease(Booton and Lindsay, 2014, Sharma et al., 2022, Moradi et al., 2025, Ardekani and Naeini, 2010, Suzuki, 2023) and chemical toxicity(Jardim et al., 2009, Halappanavar et al., 2011, Dasgupta et al., 2021, Hou et al., 2011), including respiratory-related diseases such as asthma and PAH-mediated toxicity, and so may serve as an important regulatory mechanism leading to common adverse health outcomes. miRNAs are evolutionarily conserved, small non-coding RNA, typically around 22 nucleotides long (Dong et al., 2013). Broadly, non-coding RNA have become increasingly important to understand cellular responses as they are thought to account for over 80% of the human transcriptome (Gomes et al., 2019). Although miRNAs only account for 3% of the transcriptome, they are known to be involved in many biological processes by targeting messenger RNA (mRNA) with complimentary sequences and prohibiting their translation to proteins. Through translational inhibition, mRNA degradation, and mRNA sequestering, miRNA ultimately target mRNA to prevent protein translation resulting in reduced protein levels which can have consequences on many biological processes (Dong et al., 2013) with dysregulation of miRNA being found in all human cancers (Croce, 2009, Iorio and Croce, 2012), inflammation (O’Connell et al., 2012), and respiratory diseases like asthma (Sharma et al., 2022). In various respiratory diseases like asthma, COPD, and lung cancer, miRNA have been proposed as biomarkers due to their prevalence (Sharma et al., 2022, Moradi et al., 2025). Particularly in asthma, numerous miRNAs have been detected in both childhood and adult asthma and have been characterized as mediators in airway remodeling, immune response, and inflammation (Sharma et al., 2022). There is also evidence that environmental factors, like PAH exposure, can result in dysregulated miRNA expression (Jardim et al., 2009, Halappanavar et al., 2011). Specifically, BAP was found to regulate several miRNA families associated with cancer development and altered immune functions (Halappanavar et al., 2011).

In this study, we evaluated miRNA and their predicted functional consequences after BAP exposure in an *in vitro* respiratory model of IL-13-induced asthma to better understand mechanisms contributing to unique or altered susceptibility of individuals with pre-existing airway inflammation to BAP toxicity.

## 2 Materials and methods

### 2.1 Chemicals and reagents

Cell culture media was provided by STEMCELL Technologies (Vancouver, Canada). Benzo[a]pyrene (BAP) (CAS# 50-32-8) was purchased from MRIGlobal (Kansas City, MO). Interleukin-13 (IL-13) was purchased from R&D Systems (Minneapolis, MN). Ultra pure dimethyl sulfoxide (DMSO) (CAS# 67-68-5) was purchased from VWR Chemicals (Solon, OH). Gibco^TM^ Dulbecco’s phosphate-buffered saline without calcium and magnesium ions (DPBS) was purchased from Thermo Fisher Scientific (Waltham, MA).

### 2.2 Tissue culture and treatments

Primary HBEC (Lot# 464078, Lonza, Morristown, NJ) were cultured and treated following methods previously described (Valdez et al., 2024). Briefly, primary HBEC at passage 2 were expanded to 80%–90% confluency, harvested by trypsinization, and transferred to transwell inserts (Corning, 3,470, Kennebunk, ME) for differentiation at the air–liquid interface in 24-well plates. Each well contained 500 µL of culture medium utilizing the PneumaCult^TM^ ALI medium (STEMCELL Technologies, Vancouver, Canada) prepared per manufacturer’s instructions. Cells were cultured at 37 °C and 5% CO_2_ with media changes every 48–65 h. Beginning on day 10, HBEC were differentiated in the presence of 10 ng/mL IL-13 in the cell culture media for 14 days to induce an asthmatic phenotype, as described previously (Valdez et al., 2024, Roberts et al., 2018, Malavia et al., 2008). After day 14, cells were washed with 200 µL DPBS (pH 7.0–7.3) once every 7 days in order to remove excess mucus from the apical surface. On day 25, cells were washed with DPBS then treated with either 25 µL vehicle control (1% DMSO in DPBS) or BAP (40 ug/mL) in 1% DMSO in DPBS on the apical surface for 48 h, based on prior studies (Valdez et al., 2024, Chang et al., 2019). The tissues were removed from the insert, stored in Buffer RLT (Qiagen) for miRNA isolation, and frozen at −80 °C.

### 2.3 miRNA isolation and miRNA-sequencing

Total RNA was isolated from HBEC stored in Buffer RLT (Qiagen) using TRIzol (Thermo Fisher Scientific) following the manufacturer’s protocol. RNA was quantified on a Synergy HTX plate reader equipped with a Take3 module (BioTek, Winooski, VT). Samples exposed to vehicle control and BAP in both the normal and IL-13 induced asthmatic phenotypes (n = 4) were selected for sequencing. RNA quantity was evaluated based on 280/260 ratio. RNA integrity (RIN) was assessed using an Agilent Bioanalyzer (Agilent, Santa Clara, CA) with samples having values ≥ 8.0 used for library construction and sequencing on DNBseq platform at the Beijing Genomic Institute (BGI) Americas (Cambridge, MA) with a sequencing length of 50  bp single-end reads. Following sequencing, high-quality reads were aligned to the human reference genome GRCh38. p12 using Bowtie2 and differential expression was estimated using DESeq2 platform. The differentially expressed miRNAs (q < 0.01, average read count > 5) were separated into upregulated and downregulated groups by treatment. miRNA were paired in an anti-correlated fashion with differentially expressed mRNA (q < 1 × 10^−7^, |log2FC|>0.58) collected under the same treatment conditions as previously published (Valdez et al., 2024). Differentially expressed mRNA (for each treatment were separated into upregulated and downregulated groups by treatment for integration with miRNA predicted target genes. Raw and normalized sequencing files are available online at NCBI Gene Expression Omnibus (GSE239797 for mRNA, GSE305614 for miRNA).

### 2.4 miRNA and predicted target analysis

Predicted miRNA targets were identified using the microRNA Data Integration Portal (miRDIP) (Version 5.3.0.2, Database version 5.2.3.1) and integrated with anti-correlated differentially expressed mRNA from HBEC (Valdez et al., 2024) based on published workflows (Brown et al., 2019). MiRNA-mRNA target interactions were further filtered for high-confidence predictions identified from at least 15 sources from miRDIP. Venn diagrams of significant miRNA and significant predicted mRNA targets were generated using the VennDiagram R package (Version 1.7.3). Heatmap visualizations of both significant miRNA and significant predicted mRNA targets were generated using the pheatmap R package (Version 1.0.12). The upregulated and downregulated lists of significant predicted mRNA targets for each treatment were used in MetaCore (GeneGo, Thomson Reuters, Carlsbad, CA) for pathway enrichment analysis. Statistical significance for enrichment was calculated using a hypergeometric distribution, where the *P*-value represents the probability of a particular mapping arising by chance for experimental data compared with the background, which included all genes in the human genome. miRNA predicted to target objects in networks of interest were uploaded to TransmiR v3.0(Liang et al., 2025) to identify enriched transcription factor-miRNA interactions. Statistical significance of overconnected interactions was also calculated using a hypergeometric distribution. Network interaction visualizations were generated in Cytoscape (Version 3.10.2).

## 3 Results

### 3.1 Significant miRNA and predicted targets

**Fig. 1:**
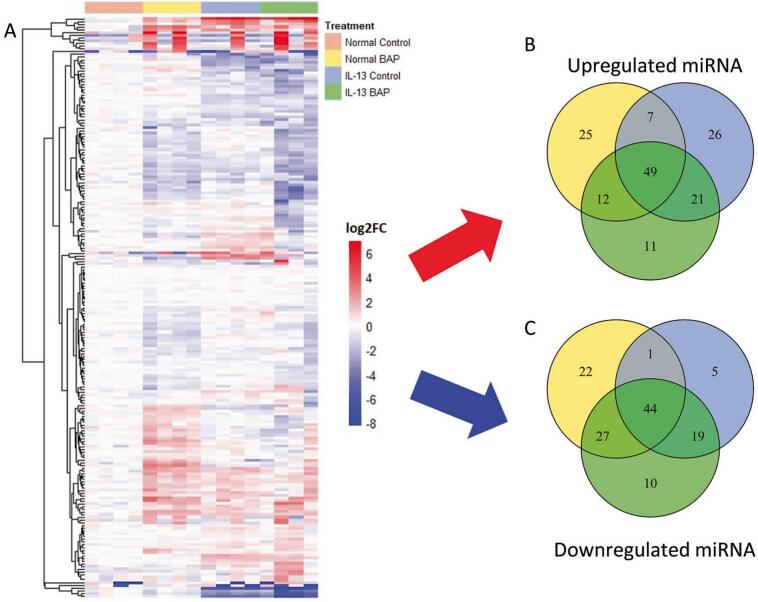
miRNA response to BAP (40 ug/mL) or Control in Normal or IL-13 phenotype HBEC. **(A)** Hierarchical clustering of significant (q < 0.01; average read count > 5) miRNA predicted to be involved in high confidence interactions in Normal BAP, IL-13 Control, and IL-13 BAP groups compared to Normal Control. Red, blue, and white represents positive, negative, and unchanged expression of miRNA compared to Normal Control, respectively. **(B)** Venn diagram of significantly increased miRNA compared to Normal Control. Yellow, blue, and green circles represent Normal BAP, IL-13 Control, and IL-13 BAP groups, respectively. **(C)** Venn diagram of significantly decreased miRNA compared to Normal Control. Yellow, blue, and green circles represent Normal BAP, IL-13 Control, and IL-13 BAP groups respectively. (For interpretation of the references to colour in this figure legend, the reader is referred to the web version of this article.)

Global miRNA and mRNA expression patterns in sequencing data were used to further investigate the role miRNA have in mediating unique responses to BAP in the IL-13-induced asthmatic phenotype HBEC. Of the 496 statistically significant (q < 0.01; average read count > 5) miRNA, 213 of those significant miRNAs were predicted to be involved in high confidence (predicted by at least 15 of the 24 tools in miRDIP) interactions. A heatmap of the significant miRNA was generated using complete hierarchical clustering analysis (Fig. 1A). Increased and decreased miRNA compared to Normal Control were organized into Venn diagrams (Fig. 1B & 1C) to compare regulation across all treatment groups. Normal phenotype HBEC exposed to BAP had 93 significantly increased miRNA transcripts (25 unique to normal BAP treated HBEC) and 94 significantly decreased miRNA transcripts (22 unique to normal BAP treated HBEC). IL-13-induced asthmatic phenotype HBEC exposed to vehicle control had 103 significantly increased miRNA transcripts (26 unique to IL-13 vehicle treated HBEC) and 69 significantly decreased miRNA transcripts (5 unique to IL-13 vehicle treated HBEC). IL-13-induced asthmatic phenotype HBEC exposed to BAP had 93 significantly increased miRNA transcripts (11 unique to IL-13 BAP treated HBEC) and 100 significantly decreased miRNA transcripts (10 unique to IL-13 BAP treated HBEC). Overall, 66 of the 213 miRNA appear in both Fig. 1B and 1C as they were significantly increased and decreased in a treatment-specific manner.

**Fig. 2:**
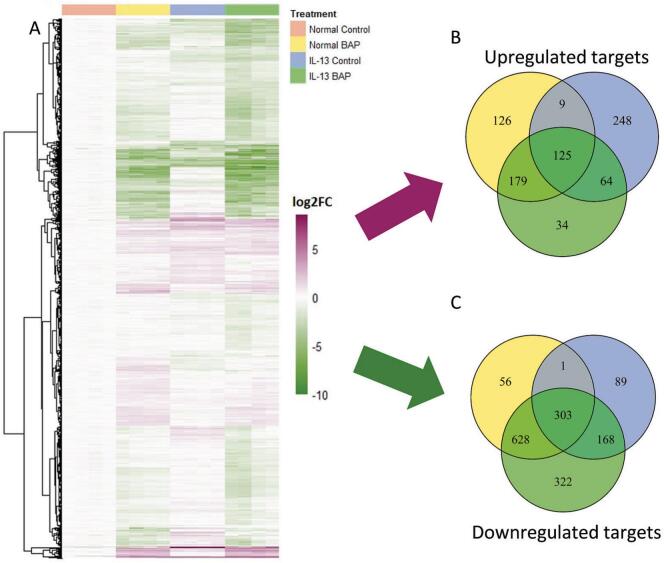
High confidence targets of miRNA in response to BAP (40 ug/mL) or Control in Normal or IL-13 phenotype HBEC. **(A)** Hierarchical clustering of significant (|log2(Fold Change)|>0.58; q < 1 × 10^−7^) high confidence targets of miRNA in Normal BAP, IL-13 Control, and IL-13 BAP groups compared to Normal Control. Purple, green, and white represent positive, negative, and unchanged expression of targets compared to Normal Control, respectively. **(B)** Venn diagram of significantly increased high confidence targets compared to Normal Control. Yellow, blue, and green circles represent Normal BAP, IL-13 Control, and IL-13 BAP groups compared to Normal Control, respectively. **(C)** Venn diagram of significantly decreased high confidence targets compared to Normal Control. Yellow, blue, and green circles represent Normal BAP, IL-13 Control, and IL-13 BAP groups compared to Normal Control, respectively. (For interpretation of the references to colour in this figure legend, the reader is referred to the web version of this article.)

To determine the functional consequences of miRNA regulation in lung cells, high-confidence miRNA gene targets predicted from miRDIP (restricted to targets predicted in at least 15 of the 24 tools) were further filtered based on mRNA significantly regulated by BAP in our lung model. We previously identified 12,275 mRNA as statistically significant (|log_2_FC|>0.58; q < 1 × 10^−7^) (Valdez et al., 2024). Since miRNA-target functional interactions typically result in down-regulation of gene targets, significant mRNA were paired in an anticorrelated fashion with the statistically significant miRNA for downstream analysis. A heatmap of the resulting 2,139 high confidence miRNA-targets was generated using complete hierarchical clustering analysis (Fig. 2A). Increased and decreased miRNA targets compared to Normal Control were organized into Venn diagrams (Fig. 2B & 2C) to compare regulation across all treatment groups. BAP-treated Normal phenotype HBEC had 439 upregulated targets (126 unique to Normal BAP treated HBEC) and 988 downregulated targets (56 unique to Normal BAP treated HBEC). Control-treated HBEC with the IL-13-induced asthmatic phenotype had 446 upregulated targets (248 unique to IL-13 Control HBEC) and 561 downregulated targets (89 unique to IL-13 control HBEC). BAP-treated HBEC with the IL-13-induced asthmatic phenotype had 402 upregulated targets (34 unique to IL-13 BAP HBEC) and 1,421 downregulated targets (322 unique to IL-13 BAP HBEC). Overall, 213 of the 2139 target mRNA appear in both Fig. 2B and 2C as they were significantly increased and decreased in a treatment-specific manner.

### 3.2 MiRNA-mediated pathway analysis

**Fig. 3:**
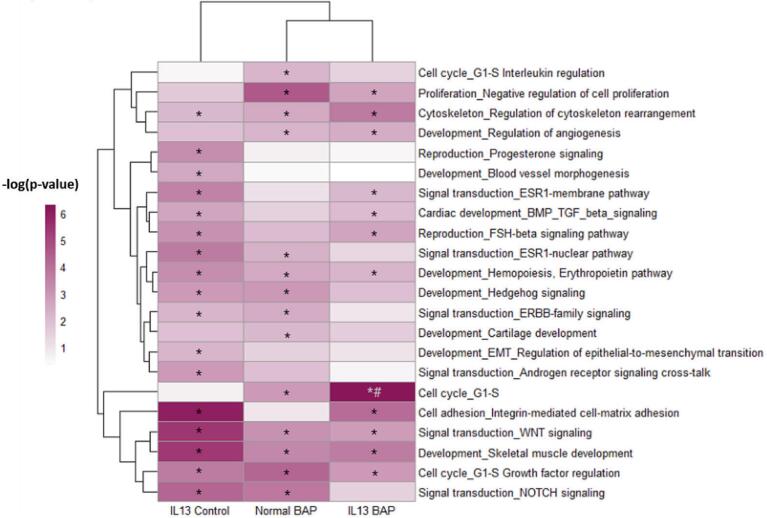
Biological processes significantly (p < 0.01) enriched in HBEC miRNA targets increased in Normal BAP, IL-13 Control, and IL-13 BAP groups compared to Normal Control. Significance increased with color shown in heatmaps. *Indicates p < 0.01 of the indicated biological process enriched in the indicated treatment group compared to Normal Control. #Indicates that the pvalue for IL-13 BAP group was at least 100-fold lower than either the Normal BAP or IL-13 Control groups suggesting a unique response of the IL-13 asthmatic phenotype to BAP treatment.

**Fig. 4:**
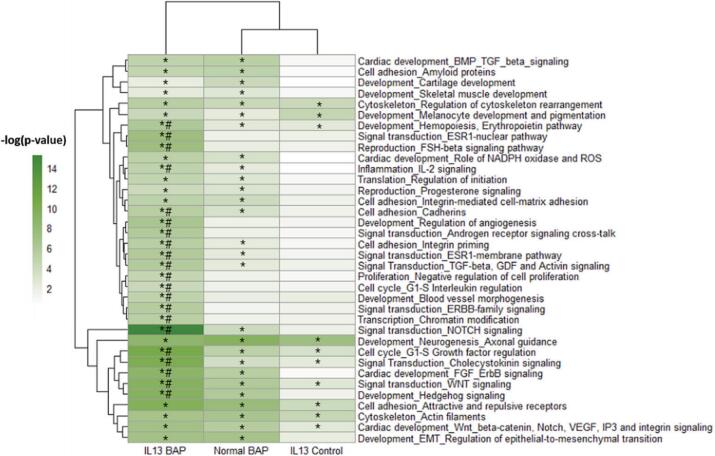
Biological processes significantly (p < 0.01) enriched in HBEC miRNA targets decreased in Normal BAP, IL-13 Control, and IL-13 BAP groups compared to Normal Control. Significance increased with color shown in heatmaps. *Indicates p < 0.01 of the indicated biological process enriched in the indicated treatment group compared to Normal Control. #Indicates that the pvalue for IL-13 BAP group was at least 100-fold lower than either the Normal BAP or IL-13 Control groups suggesting a unique response of the IL-13 asthmatic phenotype to BAP treatment.

In order to understand the downstream impacts of the significantly regulated miRNA, the up- and down-regulated high confidence target lists of the Normal BAP, IL-13 Control, and IL-13 BAP treatment groups were assessed independently by pathway analysis for enriched biological processes. Processes that were significantly (p < 0.01) regulated in at least one treatment group were incorporated into heatmap to visualize and compare miRNA functional regulation across treatment groups (Fig. 3, Fig. 4). We identified 22 processes that were significantly (p < 0.01) altered by upregulated targets of downregulated miRNA compared to Normal control cells (Fig. 3). Of those 22 processes, 14 were significantly changed in the Normal BAP group, 17 were significantly changed by the IL-13 Control group, and 12 were significantly changed by the IL-13 BAP group. In addition, 36 processes were identified as significantly (p < 0.01) altered by downregulated targets of upregulated miRNA (Fig. 4). All 36 of the processes were significant in the IL-13-induced asthmatic phenotype cells after BAP treatment, and 27 processes were significant after BAP treatment in normal cells with a subset of 10 processes also significant in the IL-13-induced asthmatic phenotype Control group. We further evaluated instances where the p-value for the IL-13 BAP group was at least 100-fold lower than either the Normal BAP or IL-13 Control groups suggesting a unique response of the IL-13 asthmatic phenotype to BAP treatment. Of the processes that met this criteria, one process, Cell cycle_G1-S, was elevated in the IL-13-BAP group (Fig. 3) and 21 processes were repressed (Fig. 4) suggesting an interaction of phenotype and chemical treatment leading to toxicity. Notably, the signal transduction pathways related to NOTCH, WNT and hedgehog signaling were the most significantly enriched pathways in the IL-13 BAP treatment group compared to the other groups.

**Fig. 5:**
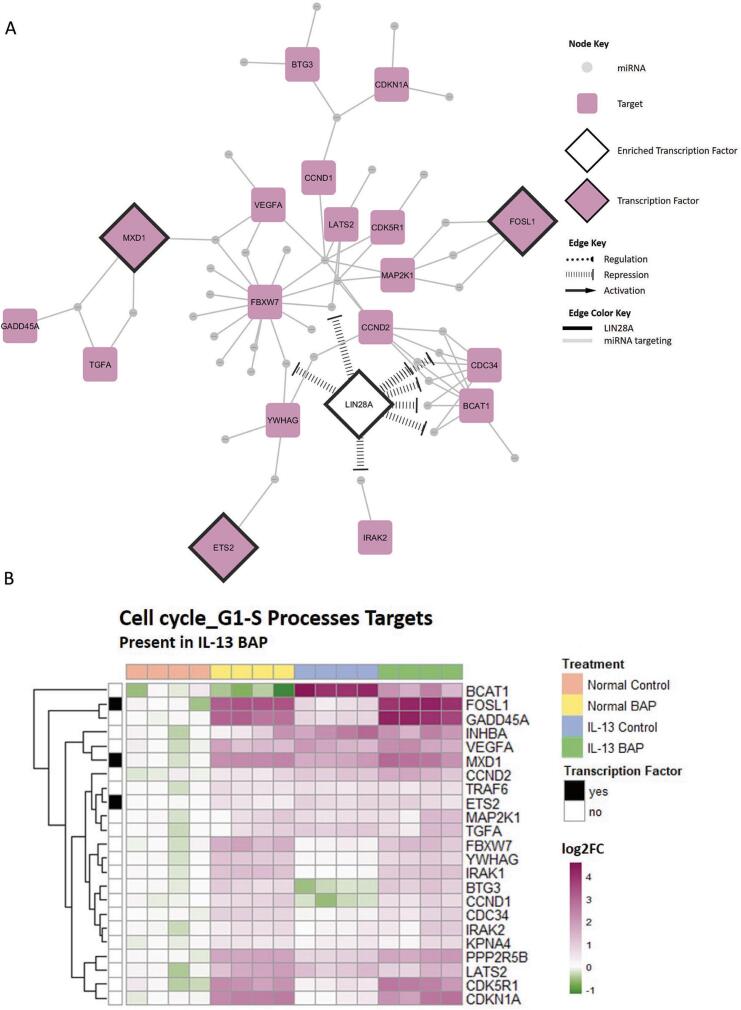
Visualization of uniquely enriched biological process related to cell cycle signaling in the IL-13 BAP group. **(A)** Network of targets unique in the IL-13 BAP group relevant in Cell cycle_G1-S, Cell cycle_G1-S interleukin regulation, and Cell cycle_G1-S Growth factor regulation biological processes. Gray circular nodes represent significant downregulated miRNA, purple round rectangle nodes represent significant upregulated targets, purple diamonds with black outlines represent upregulated targets encoding transcription factors, and white diamonds with black outlines represent enriched transcription factors predicted to target miRNA. Gray edges indicate miRNA regulating targets. Black hashed edges indicate repression of miRNA by the enriched transcription factor LIN28A. **(B)** Hierarchical clustering of increased targets in the IL-13 BAP group relevant in Cell cycle_G1-S, Cell cycle_G1-S interleukin regulation, and Cell cycle_G1-S Growth factor regulation biological processes. Purple, green, and white represent increased, decreased, and unchanged expression of targets compared to average Normal Control response. Column annotation indicates treatment group where orange, yellow, blue, and green represent Normal Control, Normal BAP, IL-13 Control, and IL-13 BAP, respectively. Row annotation indicates whether the gene encodes a transcription factor where black and white represent transcription factor and non-transcription factor, respectively. (For interpretation of the references to colour in this figure legend, the reader is referred to the web version of this article.)

**Fig. 6:**
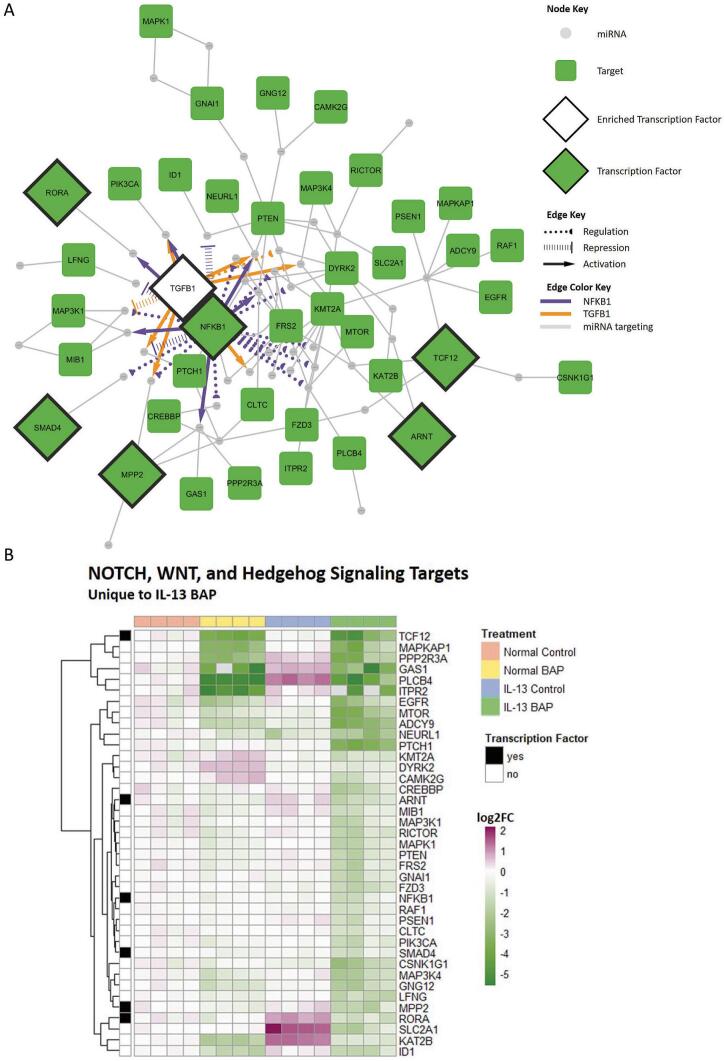
Visualization of uniquely enriched biological process related to NOTCH/WNT/Hedgehog signaling in the IL-13 BAP group. **(A)** Network of targets unique the IL-13 BAP group relevant in Signal transduction_NOTCH signaling, Signal transduction_WNT signaling, and Development_Hedgehog signaling biological processes. Gray circular nodes represent significant upregulated miRNA, green round rectangle nodes represent significant downregulated targets, green diamonds with black outlines represent downregulated targets encoding transcription factors, and white diamonds with black outline represent enriched transcription factors predicted to target miRNA. Gray edges indicate miRNA regulating targets. Orange and purple edges indicate regulation of miRNA by enriched transcription factors, TFGB1 and NFKB1, respectively. Dotted edges indicate unclear regulation, hashed edges indicate repression, and solid edges with an arrowhead indicate activation. **(B)** Hierarchical clustering of decreased targets uniquely mediated by miRNA in the IL-13 BAP group relevant in Signal transduction_NOTCH signaling, Signal transduction_WNT signaling, and Development_Hedgehog signaling biological processes. Purple, green, and white represent increased, decreased, and unchanged expression of targets compared to average Normal Control response. Column annotation indicates treatment group where orange, yellow, blue, and green represent Normal Control, Normal BAP, IL-13 Control, and IL-13 BAP, respectively. Row annotation indicates whether the gene encodes a transcription factor where black and white represent transcription factor and non-transcription factor, respectively. (For interpretation of the references to colour in this figure legend, the reader is referred to the web version of this article.)

In order to further explore the unique pathway regulation mediated by miRNA-target interactions in the IL-13-induced asthmatic phenotype after treatment with BAP, cell cycle G1-S (Fig. 5) and Notch/Wnt/Hedgehog signaling (Fig. 6) networks and target heatmaps were created. Additionally, upstream transcription factors were incorporated into the cell cycle G1-S network (Fig. 5A) and the Notch/Wnt/Hedgehog signaling network (Fig. 6A) to further assess interactions among miRNA in the network. Within the cell cycle G1-S network, LIN28A was most significantly enriched transcription factor (FDR = 2.75 × 10^−6^) and was predicted to repress 8 of the 44 miRNA (Table 1). Within the Notch/Wnt/Hedgehog signaling network, TGFB1 and NFKB1 were the top significantly enriched transcription factors (FDR < 0.01). As shown in Table 1, TGFB1 was predicted to regulate 8 of 46 miRNAs while NFKB1 was predicted to regulate 22 of 46 miRNAs in the Notch/Wnt/Hedgehog signaling processes. Overall, these data suggest that HBEC with the IL-13-induced asthmatic phenotype exposed to BAP have unique miRNA-mediated dysregulation of several processes compared to Normal BAP and IL-13 Control groups.

**Table 1:** Predicted transcription factors enriched for regulation of miRNA.

**Network Group**	**Enriched Transcription Factor**	**FDR**	**Action Type**	**microRNA targets**
Cell Cycle G1-S Processes	LIN28A	2.75E-06	Repression	hsa-let-7a, hsa-let-7c, hsa-let-7f, hsa-let-7g, hsa-let-7i, hsa-miR-107, hsa-miR-150, hsa-miR-200c
Notch/Wnt/Hedgehog Signaling	TGFB1	5.03E-04	Activation	hsa-miR-143, hsa-miR-145, hsa-miR-155, hsa-miR-199a, hsa-miR-23a, hsa-miR-424
			Repression	hsa-let-7d
			Regulation	hsa-miR-24
	NFKB1	4.11E-03	Activation	hsa-let-7b, hsa-miR-143, hsa-miR-30c, hsa-miR-130a, hsa-miR-34a
			Repression	hsa-miR-125b, hsa-miR-29b, hsa-miR-424
			Regulation	hsa-let-7d, hsa-miR-155, hsa-miR-182, hsa-miR-183, hsa-miR-193b, hsa-miR-23a, hsa-miR-25, hsa-miR-339, hsa-miR-423, hsa-miR-483, hsa-miR-532, hsa-miR-625, hsa-miR-942, hsa-miR-96

## 4 Discussion

This study investigated miRNA-mediated changes after BAP exposure in a 3D respiratory model for asthma. Mechanisms of BAP toxicity mediated by miRNAs were identified in the presence of pre-existing inflammation in an *in vitro* asthmatic phenotype through the evaluation of anti-correlated transcriptional regulation between miRNA and predicted mRNA targets. These data provide insight into the role of miRNAs mediating toxicity in susceptible individuals.

miRNAs play an important role in regulating mRNAs translation into protein. While the complex mechanism occurring between miRNAs and their target mRNAs is still being explored (Diener et al., 2024), it has been demonstrated that miRNA-target interactions result in target degradation or repression of protein translation (Wu and Brewer, 2012, Fabian et al., 2010, Bartel, 2009, Huntzinger and Izaurralde, 2011). Due to the complex role of miRNA and its relatively recent discovery, the role of miRNA as important regulators in response to chemical exposure and disease development/progression is not fully understood.

Exposure to PAHs, particularly BAP, have been demonstrated to alter expression of miRNA known to be associated with cancer development and immune function (Halappanavar et al., 2011, Huang et al., 2015). The role of miRNA in cancer development and progression has been well-studied with some studies concluding that expression of certain miRNA families are associated with poor prognosis, in particular (Booton and Lindsay, 2014, Wang et al., 2015). Furthermore, some miRNAs have been classified as tumor promoting or tumor suppressing based on the role their targets have in cancer pathogenesis and other miRNAs have been implicated specifically in human lung cancers (e.g. miR-21 and members of the let-7 family) (Booton and Lindsay, 2014, Iorio and Croce, 2012). Overall, much less is known about the role of miRNAs in non-cancer respiratory diseases and chemical toxicity in the lung; although several miRNAs identified in cancer have also been associated with asthma (Booton and Lindsay, 2014), possibly due to their roles in major cell processes such as proliferation and apoptosis. While still a broadly understudied regulator, studies have suggested that miRNA play an important role in understanding response to chemical exposures and disease development and progression.

Overall, a relatively small number of upregulated mRNA were predicted as targets of downregulated miRNA in the IL-13 BAP treated HBEC. When assessing the functional consequences of downregulated miRNA that were unique to the cells with the IL-13 induced asthmatic phenotype after treatment with BAP, there was significant enrichment of targets involved in the Cell cycle_G1-S pathway. Among the IL-13 BAP upregulated targets relevant in the Cell cycle_G1-S processes, three targets, *ETS2*, *FOSL1,* and *MXD1*, were identified as genes encoding transcription factors. ETS2 is a member of a highly conserved transcription factor family present in many animals with roles in many different biological processes (Sementchenko and Watson, 2000, Verger and Duterque-Coquillaud, 2002). Across various model organisms/viruses, ETS2, like other ETS transcription family members, have unique mechanisms including the ability to interact with DNA as both a monomer and with other proteins to illicit different responses, such as the ability to act as an oncoprotein when coupled with components of AP-1 (Wasylyk et al., 1993, Wasylyk et al., 1990). In humans, it has been demonstrated that increased expression of ETS2 has been observed in asthma patients and shown to play a critical role in pathogenesis of asthma through transcription regulation and by indirectly modulating levels of cytokines associated with allergic asthma in a murine model (Jiang et al., 2025). Similar studies have supported the role of ETS2 in inducing pulmonary inflammation (Baran et al., 2011).

*FOSL1* encodes a member of the FOS protein family which can act as a subunit of AP-1 and is thought to be a critical part of responses to environmental stimuli (Sobolev et al., 2022). In our data, we observed increased expression in the Normal BAP, IL-13 Control, and IL-13 BAP groups, with the highest average expression being in the IL-13 BAP group. It is possible that the expression patterns of *FOSL1* indicate a compounding interaction effect between IL and 13 and BAP in this system. It has been demonstrated that *FOSL1* plays an important role in response to lung inflammation (Nitkin et al., 2020) as well as an important role in tumorigenesis, with overexpression being associated with the most aggressive forms of cancer (Sobolev et al., 2022).

*MXD1* encodes a protein that plays a crucial role in the MYC/MAX/MAD network. Also known as *MAD* and *MAD1*, the protein encoded by *MXD1* competes with Myc to heterodimerize with MAX to function as a transcriptional repressor(Ayer et al., 1993). Because of the opposing and competing role Mad has compared to Myc, it is believed to repress cell growth and proliferation to favor differentiation and function as a tumor suppressor (Carroll et al., 2018, Roussel et al., 1996). The increased expression of *MXD1* indicates a potentially protective role against carcinogenesis in the IL-13 BAP treated HBEC.

We also identified enriched transcription factors upstream of miRNA predicted to regulate targets in this cell cycle process within the IL-13 BAP treated HBEC. Lin28A was predicted to be a highly enriched transcription factor that is thought to repress expression of 8 miRNA in the network (Table 1). The primary targets of Lin28A repression are members of the let-7 family (Wang et al., 2015, Piskounova et al., 2011)which are evolutionarily conserved and play important roles in many biological processes, with specific key roles in normal development (Su et al., 2012). Furthermore, overexpression of Lin28A, as well as its homolog, Lin28B, were shown to be significantly correlated with poorer overall survival, disease-free survival, progression-free survival, and relapse-free survival of cancer patients with various cancer types (Zhang et al., 2019). While significant change Lin28A expression was not observed at the 48-hour timepoint, this suggests that overexpression of Lin28A may occur at an earlier timepoint, explaining the decrease in its target miRNAs. Overall, this also suggests an increase in signaling processes related to cancer in the IL-13-induced asthmatic phenotype after BAP treatment compared to normal cells.

Overall, a larger proportion of targets from the IL-13 BAP treated HBEC had decreased expression compared to targets with increased expression associated with cell cycle regulation. When further analyzed to understand which biological processes would be impacted by the targets of upregulated miRNA, many processes were identified as significantly enriched for this group of mRNA targets. In particular, Notch, Wnt, and Hedgehog signaling processes were identified as highly significant in the IL-13-induced asthmatic phenotype after treatment with BAP. The Notch, Wnt, and Hedgehog signaling processes play crucial roles during embryonic development (Pelullo et al., 2019), but have also been implicated in cancer development (Nwabo et al., 2017). Among the IL-13 BAP downregulated targets relevant in Notch, Wnt, and Hedgehog signaling processes, six genes encoding transcription factors were identified as miRNA targets only significant in the IL-13 BAP treated HBEC: *TCF12, RORA, ARNT, NFKB1, SMAD4, and MPP2*. Regardless of miRNA-mediation, four of these genes (*SMAD4, ARNT, NFKB1,* and *MPP2*) were uniquely differentially expressed in the IL-13 BAP group only suggesting an important role in mediating BAP toxicity in the asthmatic model compared to normal bronchial epithelial cells.

*SMAD4* encodes a member of the Smad family of transcription factors and is believed to play a role in inflammation as well as function as a tumor suppressor. It has been demonstrated that reduced *SMAD4* expression is common in human non-small cell lung cancer (Haeger et al., 2016). Loss of both *SMAD4* and *PTEN,* which was also predicted to be uniquely regulated in the IL-13 BAP treated HBEC, has also been shown to promote lung tumor growth and metastasis (Liu et al., 2015). Furthermore, *SMAD4* deficient cell lines demonstrated reduced capability for DNA repair (Haeger et al., 2016). Because reduction in *SMAD4* expression was only observed in the IL-13-asthmatic phenotype after BAP treatment, there may be a unique risk for accumulation of DNA damage and other mutations after exposure to BAP that is not observed in cells with a normal phenotype. Similarly, *MPP2* encodes the FoxM1 transcription factor which is predominately thought to have a role in cell proliferation and have an important role in carcinogenesis (Kalin et al., 2011). Due to its role in cell proliferation, deletion of FOXM1 in other tissues has been shown to prevent progression of the cell cycle (Kalin et al., 2011) which limits the tissue’s ability to adequately respond to injury.

*ARNT* encodes a protein that functions as the nuclear translocator of ligand-bound aryl hydrocarbon receptor (AhR), which is relevant in the IL-13 BAP treatment group, as BAP is a well-established AhR ligand (Moorthy et al., 2015, Shiizaki et al., 2017). Additionally, ARNT is known to heterodimerize with HIF-1a, which is part of the transcription factor hypoxia-inducible factor-1. Through both roles, ARNT acts as an important heterodimer of master transcription regulators. In an animal model study, it was demonstrated that conditional *ARNT* knockdown resulted in reduced airway inflammatory response due to HIF’s role in allergic airway inflammation (Huerta-Yepez et al., 2011). In addition, studies in mice with combustion-derived particulate matter, which are known to include PAHs, found that mechanisms of oxidative stress and inflammation in lung that promote pulmonary remodeling were mediated by AhR (Harding et al., 2021). Although reduction in *ARNT* may reduce certain pathological features of asthma, it can also inhibit the beneficial features of HIF-1. For example, it has been demonstrated that HIF-1a, at optimal levels, is beneficial in mediating epithelial cell response to injury (Ahmad et al., 2012). Through reduced expression of *ARNT*, HIF-1 transcription regulation would be decreased, potentially resulting in loss of ability to respond to epithelial injury. This suggests that decreased expression of *ARNT* observed in the IL-13-induced asthmatic phenotype after treatment with BAP results in a unique diminished capacity to adequately respond to injury compared to normal cells treated with BAP.

*NFKB1* encodes two subunits of the NF-kB transcription factor family, p105 and p50, and plays an important role in the NF-kB pathway. The NF-kB pathway is well studied and known to play an important role in development and regulation of innate and adaptive immunity (Aggarwal et al., n,d.). Dysregulation of the NF-kB pathway, broadly, can lead to disruption of homeostasis and has been associated with various inflammatory diseases, including airway hyperresponsiveness, asthma, and cancer (Yu et al., 2020, Almowallad et al., 2022). In asthma, NF-kB is believed to have an important role in disease pathogenesis and has been demonstrated to be activated in asthmatic airways (Serasanambati and Chilakapati, 2016). However, we only observed a significant decrease in expression of the NF-kB subunit, *NFKB1*, in the IL-13-induced asthmatic phenotype after treatment with BAP. The observed decreased expression of *NFKB1*, has been reported to mediate human lung cancer pathogenesis in which expression of *NFKB1* was significantly decreased in human lung tumor tissues compared to normal tissues from the same patients and was further shown to directly mediate lung cancer growth (Sun et al., 2016). The unique inhibition of *NFKB1* in the IL-13 BAP treated HBEC suggests that individuals with pre-existing inflammation-based lung disease may respond uniquely to chemical insult resulting in change of processes related to airway inflammation and cancer signaling pathways.

In addition to its downstream role, NFKB1 was predicted to have an upstream role, regulating nearly half of the miRNAs predicted to regulate unique IL-13 BAP targets involved in the Notch/Wnt/Hedgehog signaling pathway. Notably, NFKB1 was predicted to target several miRNAs that targeted other genes encoding transcription factors in the network such as *MPP2, SMAD4,* and *RORA.* It was also predicted to potentially regulate miRNAs that target itself, potentially resulting in a regulatory feedback loop.

Additionally, *RORA* was differentially expressed in both IL-13 phenotype groups; however, expression was increased in the IL-13 Control group and decreased in the IL-13 BAP group compared to normal control cells. No significant *RORA* expression was observed in the Normal BAP group, suggesting unique inhibition of the gene in the presence of the IL-13 phenotype after BAP exposure. While typically thought of as metabolism and circadian rhythm gene transcriptional regulator (Jetten, 2009), increased *RORA* expression has been demonstrated to play a significant role in airway inflammation, particularly the induction of the Th2 cytokines important in allergic asthma (Jaradat et al., 2006). Alternatively, a study in non-small cell lung cancer tissues demonstrated the tumor-suppressing role of *RORA* in which decreased expression of *RORA* mRNA was associated with a poorer prognosis (Xian et al., 2022). Consistent with what we observed in IL-13 BAP treated HBEC, this suggests a potentially unique risk compared to normal phenotype HBEC exposed to BAP.

In addition to NFKB1, TGFB1 was identified from transcription factor analysis as another significantly enriched protein that may be responsible for regulation of miRNAs involved in the Notch/Wnt/Hedgehog signaling network. While TGFB1 is not a transcription factor itself, it is a cytokine involved in the regulation of signaling via SMADs and has extensive crosstalk with the Wnt signaling pathway (Luo, 2017). TGFB1 is predominately predicted to activate the miRNA it targets in this network, suggesting that TGFB1 may have increased expression prior to 48 h in the IL-13 BAP treated HBEC. Increased expression of TGFB1 would be consistent with what has previously been reported in asthma patients (Saito et al., 2018, Redington et al., 1997, Vignola et al., 1997). Furthermore, increased expression of TGFB1 was also observed in mice lung after oral exposure (Labib et al., 2012). The identification of TGFB1 as an enriched regulator of miRNA in the Notch/Wnt/Hedgehog signaling pathway, as well as reported increased expression in response to asthma as well as BAP exposure, suggests that TGFB1 may play an important role upstream and help explain the miRNA-mediated impacts observed in this study.

## 5 Conclusion

Overall, this study further explores the unique risk and modulation of BAP-induced toxicity in the presence of pre-existing disease-related inflammation and allowed us to investigate how pre-existing disease alters response to environmental stimuli. Prior studies found that lung cells treated with BAP had unique transcriptional signatures in the IL-13 induced asthmatic phenotype compared to normal cells showing a decrease in barrier integrity and immune response and an increase in processes related to mucus production, goblet cell hyperplasia and type 2 asthmatic inflammation. Based on global miRNAseq analysis, in this study we were able to identify the potential role of miRNAs that are regulated in response to pre-existing inflammation-based disease and chemical BAP exposure. Our data show unique regulation of miRNA in cells with the IL-13 phenotype related to inflammatory response and cell cycle signaling, indicating an important role for miRNA signaling mediating the signaling processes associated with airway disease and chemical toxicity within a susceptible population. Future studies will explore the role of individual miRNAs mediating adverse outcomes and toxicity in lung cells.

## CRediT authorship contribution statement

**Reese M. Valdez:** Conceptualization, Methodology, Formal analysis, Investigation, Data curation, Writing – original draft, Writing – review & editing, Visualization. **Yvonne Chang:** Methodology, Writing – review & editing. **Jamie M. Pennington:** Methodology, Investigation, Resources, Writing – review & editing. **Susan C. Tilton:** Conceptualization, Data curation, Writing – review & editing, Supervision, Funding acquisition.

## Funding

This project was supported by the National Institute of Environmental Health Sciences under Award Numbers P42 ES016465, T32 ES007060 and P30 ES030287, and, in part, by the Oregon Agricultural Experiment Station with funding from the Hatch Act capacity funding program, award numbers NI25HFPXXXXXG022 and NI25HMFPXXXXG029, from the USDA National Institute of Food and Agriculture.

## Declaration of competing interest

The authors declare that they have no known competing financial interests or personal relationships that could have appeared to influence the work reported in this paper.
